# Textbook on the Therapeutic Action of Light

**Published:** 1910-11

**Authors:** 


**Textbook on the Therapeutic Action of Light.** By **Corydon Eugene Rogers**, M.D., formerly Demonstrator of Anatomy in the University of New York. Small octavo, pp. 323. Illustrated. • Published by the author.

In this book the author has presented his experience in the employment of light as a therapeutic agent in the treatment of vaiious diseases. By the aid of a specially constructed lamp of not less than 600 candle power, controlled by special reflectors, he has been able to produce what he calls new rays, or rho rays, which, when projected upon the human body, not only penetrate but pass entirely through the tissues, including the bones." If the writer has succeeded in relieving the many diseases he describes he is to be congratulated. A test will be to buy the book, read it and apply its methods in treatment. J. A. R.

